# Shoulder and Neck Balance in Adolescent Idiopathic Scoliosis: Which Radiographic Indices are Reliable and Practical?

**DOI:** 10.5704/MOJ.2403.007

**Published:** 2024-03

**Authors:** QDN Vo, HHH Nguyen, HT Nguyen, BN Pham, TK Truong

**Affiliations:** 1 Department of Pediatric Orthopaedic, Hospital for Traumatology and Orthopedics, Ho Chi Minh City, Vietnam; 2 Department of Orthopaedic, Tay Nguyen University, Buon Ma Thuot, Vietnam

**Keywords:** clavicle rib intersection distance, clavicle chest cage angle difference, T1 tilt angle, difference of outer shoulder height, neck tilt angle

## Abstract

**Introduction:**

Deformities of the spine and thorax in adolescent idiopathic scoliosis affect appearance. They are a cause of inferiority, affecting psychological well-being and the social life of the patients. To contribute to curve evaluation, planning in curve correction, and improving the post-operative aesthetics, many studies on the correlation between appearance and radiography in the assessment of shoulder and neck balance have been reported recently. In general, these studies did not clarify which indices are required to evaluate shoulder and neck balance. This study aimed to learn about indices to assess shoulder and neck balance in adolescent idiopathic scoliosis in correlation between clinical appearance and radiography.

**Materials and methods:**

This observational study recruited 50 patients with adolescent idiopathic scoliosis who were 12 to 18 years of age with Cobb angle >10°. Based on Pearson correlation coefficient, radiographic parameters such as coracoid height difference (CHD), clavicle rib intersection distance (CRID), clavicle angle (CA), clavicle chest cage angle difference (CCAD), and T1 tilt angle were evaluated in correlation with clinical shoulder and neck balance by difference of inner shoulder height (SHi), difference of outer shoulder height (SHo), and neck tilt angle.

**Results:**

SHi was moderately correlated with T1 tilt angle (r [hereafter] = 0.45), CA (0.47), and CHD (0.57), high-moderately correlated with CRID (0.64), very-highly correlated with CCAD (0.84). SHo was moderately correlated with T1 tilt angle (0.43), highly correlated with CHD (0.60), CA (0.63), and CRID (0.72), and very-highly correlated with CCAD (0.89). T1 tilt angle was high-moderately correlated with neck tilt angle (0.76). The correlation coefficients between clinical and radiographic shoulder and neck balance according to sex, BMI, type of main curve, severity of main curve did not change significantly.

**Conclusion:**

There was a very high correlation between SHo (shoulder tilt) and CCAD (0.89); the correlation between SHo and CRID was high-moderate (0.72), but CRID is easier than CCAD to evaluate on radiographs. On the other hand, T1 tilt angle, which is the easiest radiographic parameter to evaluate, had a high-moderate correlation with neck tilt angle (0.76) but a moderate correlation with SHo (0.43).

## Introduction

Scoliosis is an abnormal curvature of the spine in the frontal plane, with a C or S shape. This curve is called scoliosis when the angle (measured by Cobb’s method) is greater than 10°^1,2^. Scoliosis is divided into different types based on its causes, such as congenital vertebral defects, neuromuscular diseases, and syndromes (e.g, Marfan, Arnold Chiari, etc.). When the reason cannot be found, it is called idiopathic scoliosis^3^.

The deformities of the spine and thorax in idiopathic scoliosis affect the patient’s appearance and occur mainly in adolescents, especially in girls. Therefore, it is a cause of inferiority, affecting psychological well-being and the social life of the patients. Especially, neck and shoulder balance are an interest for both family and surgeons. Indeed, Orvomaa *et al*^4^ studied 208 adolescent idiopathic scoliosis (AIS) postoperative patients and found that 38% of patients were pessimistic about the future, and 21.6% of patients felt miserable with their lives due to cosmetic concerns. By surgical assessment of the proximal thoracic curve in AIS, Smyrnis *et al*^5^ showed that 28.6% of patients were not satisfied with shoulder imbalance post-operatively. Zhang *et al*^6^ determined a 25% incidence of post-operative shoulder imbalance after analysing 26 separate studies. Furthermore, Kwan *et al*^7^ suggested that neck and shoulder balance should not be forgotten as a measurement, especially in patients with Lenke II.

To contribute to curve evaluation, planning in curve correction and to improve the post-operative aesthetics, many studies on the correlation between appearance and radiography in the assessment of shoulder and neck balance have been reported recently. Qiu *et al*^8^ reported that none of the radiological indices had a very high correlation (r>0.8) with clinical appearance in the assessment of shoulder balance. Yang *et al*^9^ showed no clinically high moderate correlation between anterior and posterior shoulder balance; there were no radiographic indices that were very highly correlated (r>0.8) with clinical appearance. When studying 85 patients with AIS, Yagi *et al*^10^ showed post-operative shoulder imbalance in 25% of patients and a significant correlation between post-operative shoulder girdle’s height difference with centre chest cage line angle on both sides (odds ratio=5.10; P=0.01). Kwan *et al*^11^ found a low clinical correlation between neck balance and shoulder balance in 89 AIS patients. Meanwhile, Zuckerman *et al*^12^ showed a lack of consensus among surgeons in the clinical and radiological assessments of shoulder balance pre-operatively. In general, these studies did not clarify which indices are required to evaluate shoulder and neck balance.

This study aimed to learn about the indices that can be applied to assess shoulder and neck balance in AIS in the correlation between clinical appearance and radiography.

## Materials and Methods

This observational study included patients with AIS who were 11 to 18 years of age (recommendation from SRS) with Cobb angle >10° (measured by Cobb’s method)^1^, both non-operated cases and pre-operated cases from January 2020 to September 2020. Patients were excluded from this study if neuromuscular disorders or syndromes were not ruled out. Finally, 50 patients were cross-sectionally recruited by convenience sampling.

This study was approved by Ethics Committee in Biomedical Research of UMP (University of Medicine and Pharmacy at Ho Chi Minh City – Vietnam) with the decision number 398/DHYD-HDDD signed on August 20th, 2019. Purpose and procedure of the study were informed to the participants and then consent forms were voluntarily signed.

**Fig 1:**
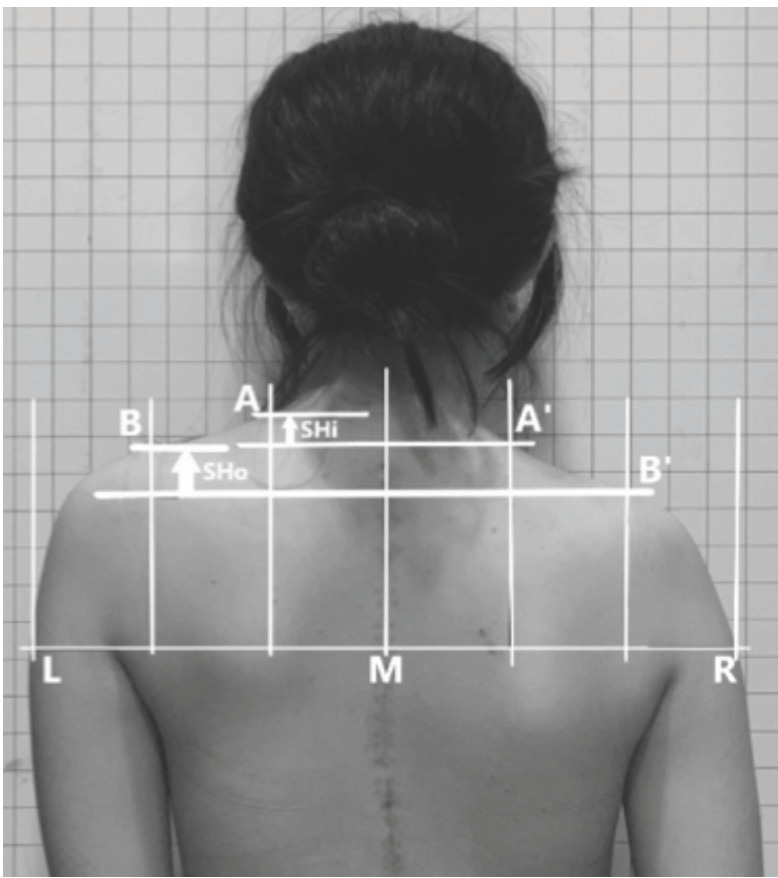
Clinical evaluation of shoulder balance (L: left, M: midpoint of the neck, R: right, SHi: inner shoulder height, SHo: outer shoulder height).

**Fig 2:**
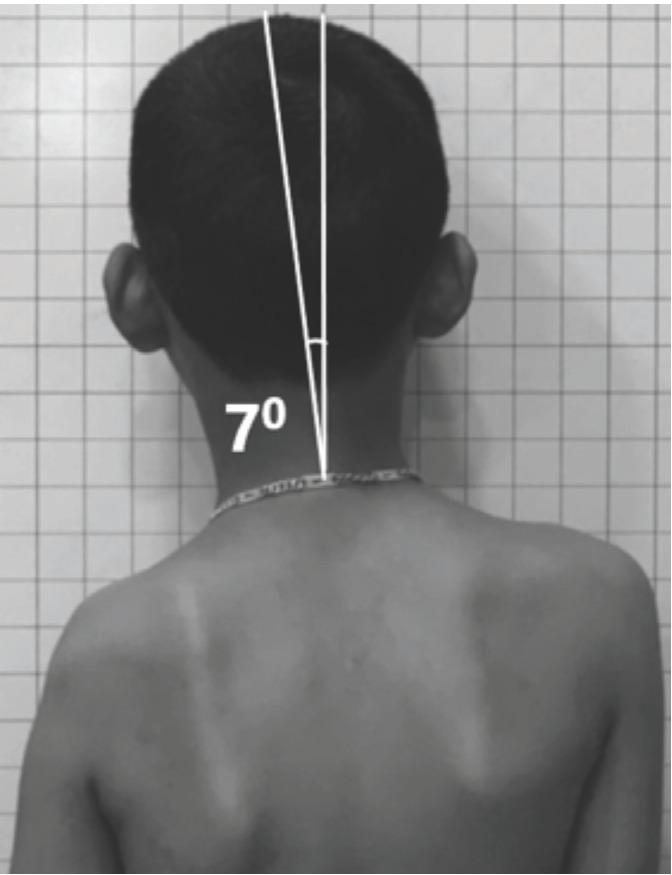
Clinical evaluation of neck balance (neck tilt angle).

For clinical evaluation of shoulder and neck balance, photos of the patients were taken in a private room with a family member next to him or her. The patient was standing in front of a panel with a grid size of 2.5cm × 2.5cm. To limit errors due to projection angle, the camera’s height was the average of heights of both shoulders and distance between the camera and the patient was 1.2m. Shoulder height (SH) (Fig. 1) was determined by the horizontal line through the higher axilla that intersects the arms on the left and right and the plumb line through the midpoint of the neck on each photo intersecting this horizontal line at M. The trisection lines of M left and M right intersected the shoulders at A, B (left) and A’, B’ (right). The difference between the heights of A and A’ was defined as the inner shoulder height (SHi), and the difference between B and B’ was defined as the outer shoulder height (SHo)^8^. SH was classified as mild (<1cm), moderate (≥1cm but <2cm), and severe (≥2cm). The angle of neck tilt (Fig. 2) was determined by the intersection of the vertical line and the longitudinal axis of the neck. This angle was defined as neck tilt if ≥5°.

**Fig 3:**
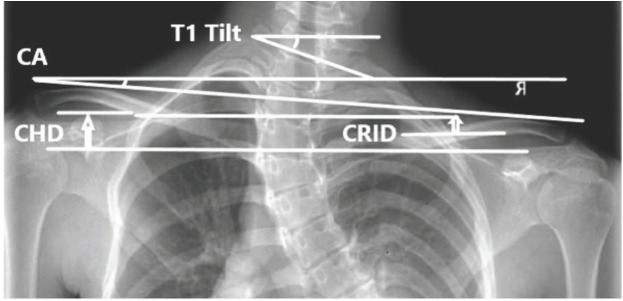
Radiographic parameters of neck-shoulder balance (CA: clavicle angle, CHD: coracoid height difference, CRID: clavicle rib intersection distance).

**Fig 4:**
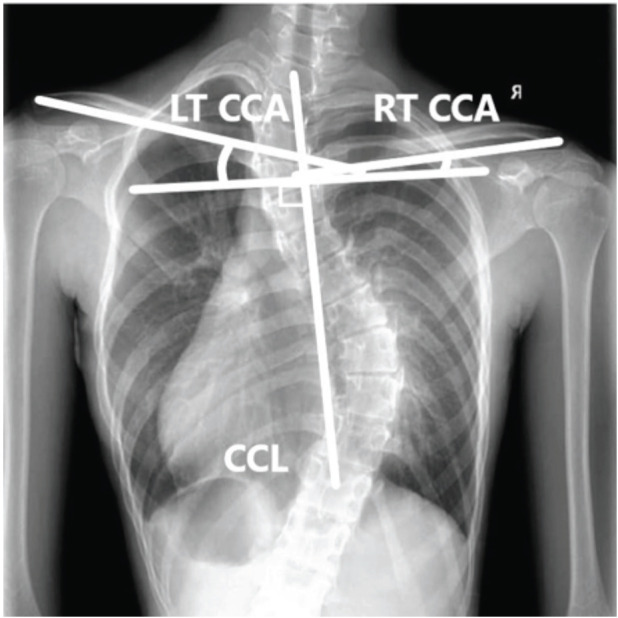
Clavicle chest cage angle difference was determined by the difference of the left clavicle chest cage angle and right clavicle chest cage angle (CCA: clavicle chest cage angle, CCL: center chest cage line, LT: left, RT: right).

For radiographic assessment of neck and shoulder balance, patients stood, looking straight ahead with their arms straight along to trunk. Coracoid height difference (CHD) was defined as the height difference between the coracoid processes. CHD was assessed by tracing a horizontal line at the upper margin of each and measuring the difference (in cm). Clavicle rib intersection distance (CRID) was defined as the height difference between the horizontal line at the level of intersection between the clavicle and the second rib (measured in cm). Clavicle angle (CA) was defined as the angle between the line connecting the lateral end of both clavicles with the horizontal plane (measured in degrees). T1 tilt angle was defined as the angle between the horizontal line and the line through the upper endplate of T1. Positive T1 tilt was represented by the angulation of the upper endplate of T1 to the horizontal line with the left proximal vertebral body up and right lower vertebral body down (Fig. 3). Clavicle chest cage angle difference (CCAD)^13^ was measured by a line (centre chest cage line) drawn from the centroid of T1 to the centroid of T12; if the 13th rib existed, then the line was drawn to the centroid of T13. A line was drawn perpendicular to the first line. Another line was then drawn from the middle of the proximal end of the clavicle to the middle of the distal end of both right and left clavicles. The angle formed by the intersection of these lines represented the clavicle chest cage angle. CCAD was then measured as the subtraction of the values of the left clavicle chest cage angle from the corresponding values of the right clavicle chest cage angle (Fig. 4).

Then, clinical images and radiographs of the entire spine were recorded and measured with AutoCAD 2019 software. Each clinical and radiographic imaging parameter was measured twice, taking the average value of the measurements; if measurements differed by more than 5%, then they were taken again. The data after the first and second measurement were analysed again; if the first and second measurements on the same variable had r≥0.95, then they were recorded. The data were analysed using the statistical software SPSS 19.0 [IBM Corp., Armonk, NY, United States] and Stata 14.0 [StataCorp., College Station, TX, United States]. The correlation between two quantitative variables was based on Pearson’s correlation coefficient (r). The degree of correlation was evaluated as follows: r<0.2 was called uncorrelated; 0.2 to < 0.4 was low; 0.4 to < 0.6 was moderate; 0.6 to <0.8 was high-moderate; 0.8 to < 1 was very-high; and = 1 was a perfect correlation. On the other hand, the variables of demographic data as age, sex, weight, height, BMI, type of main curve (thoracic or thoracolumbar/lumbar), severity of main curve (mild <25°, moderate 25-45°, severe >45°) were analysed in relation to shoulder and neck balance in correlation between clinical appearance and radiography. χ^2^ test and t-test were used for significance.

## Results

Among 50 patients with AIS 12 to 18 years of age, 74% were female (37/50 patients), and 26% were male (13/50 patients); the mean height was 151±9.2cm; the mean weight was 41.3±6.7 kg; and the mean BMI was 18.1±2.3 with 65.9% underweight (BMI <18.5), 34.1% healthy weight (BMI 18.5 – 24.9). In total, 27 patients were diagnosed with main thoracic curve (54%), 23 patients with main thoracolumbar/lumbar curve (46%); the Cobb angle of the main curve was mild, moderate, and severe in 6%, 12%, and 82% of patients, respectively.

Higher right shoulder accounted for 80% of cases, and higher left shoulder accounted for 20%. SHi was mild, moderate, and severe in 64.0%, 30.0%, and 6.0% of patients, respectively; the mean SHi was 0.80±0.60cm. SHo was mild, moderate, and severe in 44.0%, 30.0%, and 26.0% of patients, respectively; the mean SHo was 1.40±0.88cm (Table I). 9/50 patients had a neck tilt angle ≥5°, accounting for 18% with a mean neck tilt angle of 7.5° (5.1°-9.2°). The neck tilt angle had a moderate correlation with SHi and SHo (r=0.48 and 0.45, respectively) (Table II).

**Table I:** Shoulder height difference.

	SH	n = 50
SHi	Mild, %	32 (64.0)
	Moderate, %	15 (30.0)
	Severe, %	3 (6.0)
	Mean ± SD, cm	0.8±0.6
SHo	Mild, %	22 (44.0)
	Moderate, %	15 (30.0)
	Severe, %	13 (26.0)
	Mean±SD, cm	1.4±0.9

SD: Standard deviation; SH: Difference of shoulder height; SHi: Difference of inner shoulder height; SHo: Difference of outer shoulder height.

**Table II:** Clinical correlation between shoulder and neck balance.

Neck balance	SHi		SHo	
Neck tilt angle	r	P	r	P
	0.48	< 0.01	0.45	< 0.01

SHi: Difference of inner shoulder height; SHo: Difference of outer shoulder height.

Radiographic parameters of shoulder balance include mean CHD of 1.2±0.84 cm (0.03cm – 2.96cm), mean CRID of 1.0 ± 0.67 cm (0.09cm – 2.63cm), mean CA of 6.3±3.77° (0.3°16.1°), mean T1 tilt angle of 4.7±4.61° (0.0° - 23.2°), and mean CCAD of 10.1±6.88° (1.2° – 26.7°) (Table III).

**Table III:** Radiographic parameters of shoulder balance.

Radiographic parameter	Mean±SD	Range
CHD in cm	1.2±0.9	0.0 – 3.0
CRID in cm	1.0±0.7	0.1 – 2.6
CA in °	6.3±3.8	0.3 – 16.1
T1 tilt angle in °	4.7±4.6	0.0 – 23.2
CCAD in °	10.1±6.9	1.2 – 26.7

CA: Clavicle angle; CCAD: Clavicle chest cage angle difference; CHD: Coracoid height difference; CRID: Clavicle rib intersection distance.

Correlation coefficients between SH (SHi and Sho) and radiographic parameters of shoulder balance are shown in (Table IV). SHi had a moderate correlation with T1 tilt angle (r=0.45), CA (r=0.47), and CHD (r=0.57). SHi had a high-moderate correlation with CRID (r=0.64) and a very high correlation with CCAD (r=0.84). SHo had a moderate correlation (r=0.43) with T1 tilt angle. SHo had a high-moderate correlation with CHD (r=0.60), CA (r=0.63), and CRID (r=0.72). SHo had a very-high correlation with CCAD (r=0.89). These coefficients according to sex, BMI, type of main curve, severity of main curve did not change significantly (Table V, Table VI, Table VII and Table VIII).

**Table IV:** Correlation between clinical appearance and radiography in the evaluation of shoulder balance.

Radiographic parameter	SHi		SHo	
	r	P	r	P
CHD in cm	0.57	<0.01	0.60	<0.01
CRID in cm	0.64	<0.01	0.72	<0.01
CA in °	0.47	<0.01	0.63	<0.01
T1 tilt angle in °	0.45	<0.01	0.43	<0.01
CCAD in °	0.84	<0.01	0.89	<0.01

CA: Clavicle angle; CCAD: Clavicle chest cage angle difference; CHD: Coracoid height difference; CRID: Clavicle rib intersection distance; SHi: Difference of inner shoulder height; SHo: Difference of outer shoulder height.

**Table V:** Correlation between clinical appearance and radiography in the evaluation of shoulder balance according to sex.

Radiographic parameter	SHi				SHo			
	Female		Male		Female		Male	
	r	P	r	P	r	P	r	P
CHD in cm	0.59	<0.01	0.53	<0.01	0.60	<0.01	0.62	<0.01
		P>0.05				P>0.05		
CRID in cm	0.66	<0.01	0.63	<0.01	0.73	<0.01	0.69	<0.01
		P>0.05				P>0.05		
CA in °	0.50	<0.01	0.46	<0.01	0.64	<0.01	0.63	<0.01
		P>0.05				P>0.05		
T1 tilt angle in °	0.46	<0.01	0.45	<0.01	0.42	<0.01	0.43	<0.01
		P>0.05				P>0.05		
CCAD in °	0.85	<0.01	0.81	<0.01	0.90	<0.01	0.89	<0.01
		P>0.05				P>0.05		

CA: Clavicle angle; CCAD: Clavicle chest cage angle difference; CHD: Coracoid height difference; CRID: Clavicle rib intersection distance; SHi: Difference of inner shoulder height; SHo: Difference of outer shoulder height.

**Table VI:** Correlation between clinical appearance and radiography in the evaluation of shoulder balance according to BMI.

Radiographic parameter	SHi				SHo			
	Underweight		Healthy weight		Underweight		Healthy weight	
	r	P	r	P	r	P	r	P
CHD in cm	0.60	<0.01	0.54	<0.01	0.63	<0.01	0.59	<0.01
		P>0.05				P>0.05		
CRID in cm	0.65	<0.01	0.64	<0.01	0.74	<0.01	0.69	<0.01
		P>0.05				P>0.05		
CA in °	0.51	<0.01	0.46	<0.01	0.64	<0.01	0.62	<0.01
		P>0.05				P>0.05		
T1 tilt angle in °	0.45	<0.01	0.45	<0.01	0.42	<0.01	0.44	<0.01
		P>0.05				P>0.05		
CCAD in °	0.84	<0.01	0.84	<0.01	0.90	<0.01	0.88	<0.01
		P>0.05				P>0.05		

CA: Clavicle angle; CCAD: Clavicle chest cage angle difference; CHD: Coracoid height difference; CRID: Clavicle rib intersection distance; SHi: Difference of inner shoulder height; SHo: Difference of outer shoulder height.

**Table VII:** Correlation between clinical appearance and radiography in the evaluation of shoulder balance according to type of main curve.

Radiographic parameter	SHi				SHo			
	Thoracic		Thoracolumbar/Lumbar		Thoracic		Thoracolumbar/Lumbar	
	r	P	r	P	r	P	r	P
CHD in cm	0.58	<0.01	0.56	<0.01	0.61	<0.01	0.61	<0.01
		P>0.05				P>0.05		
CRID in cm	0.65	<0.01	0.63	<0.01	0.72	<0.01	0.72	<0.01
		P>0.05				P>0.05		
CA in °	0.49	<0.01	0.44	<0.01	0.64	<0.01	0.61	<0.01
		P>0.05				P>0.05		
T1 tilt angle in °	0.45	<0.01	0.46	<0.01	0.41	<0.01	0.44	<0.01
		P>0.05				P>0.05		
CCAD in °	0.82	<0.01	0.84	<0.01	0.87	<0.01	0.91	<0.01
		P>0.05				P>0.05		

CA: Clavicle angle; CCAD: Clavicle chest cage angle difference; CHD: Coracoid height difference; CRID: Clavicle rib intersection distance; SHi: Difference of inner shoulder height; SHo: Difference of outer shoulder height.

**Table VIII:** Correlation between clinical appearance and radiography in the evaluation of shoulder balance according to severity of main curve.

Radiographic parameter	SHi						SHo					
	Mild		Moderate		Severe		Mild		Moderate		Severe	
	r	P	r	P	r	P	r	P	r	P	r	P
CHD in cm	0.57	<0.01	0.56	<0.01	0.57	<0.01	0.61	<0.01	0.61	<0.01	0.59	<0.01
			P > 0.05						P > 0.05			
CRID in cm	0.64	<0.01	0.63	<0.01	0.64	<0.01	0.70	<0.01	0.70	<0.01	0.73	<0.01
			P > 0.05						P > 0.05			
CA in °	0.49	<0.01	0.47	<0.01	0.47	<0.01	0.64	<0.01	0.62	<0.01	0.62	<0.01
			P > 0.05						P > 0.05			
T1 tilt angle in °	0.44	<0.01	0.46	<0.01	0.44	<0.01	0.44	<0.01	0.44	<0.01	0.43	<0.01
			P > 0.05						P > 0.05			
CCAD in °	0.83	<0.01	0.83	<0.01	0.84	<0.01	0.87	<0.01	0.91	<0.01	0.89	<0.01
			P > 0.05						P > 0.05			

CA: Clavicle angle; CCAD: Clavicle chest cage angle difference; CHD: Coracoid height difference; CRID: Clavicle rib intersection distance; SHi: Difference of inner shoulder height; SHo: Difference of outer shoulder height.

T1 tilt angle had a high-moderate correlation (r = 0.76) with neck tilt angle. This correlation between clinical appearance and radiography of neck balance according to sex, BMI, type of main curve, severity of main curve did not change significantly (Table IX).

**Table IX:** Correlation between clinical appearance and radiography of neck balance according to sex, BMI, type of main curve, severity of main curve.

Neck tilt angle	T1 tilt angle in °		
	r		P
Sex			
Female	0.75		<0.01
Male	0.76		<0.01
		P > 0.05	
BMI			
Underweight	0.76		<0.01
Healthy weight	0.77		<0.01
		P>0.05	
Type of main curve			
Thoracic	0.77		<0.01
Thoracolumbar/Lumbar	0.74		<0.01
		P>0.05	
Severity of main curve			
Mild	0.75		<0.01
Moderate	0.75		<0.01
Severe	0.76		<0.01
		P>0.05	

## Discussion

T1 tilt angle has been used to assess shoulder balance, but recent studies have shown that this correlation is not significant. When studying 33 children with AIS who needed surgery, Bago *et al*^14^ showed that clinical shoulder balance was correlated with T1 tilt angle (r=0.54) but not as much as with CHD (r=0.96) and CRID (r=0.93). When studying shoulder balance between clinical appearance and radiography in normal adolescents, Akel *et al*^15^ also noted that the T1 tilt angle had a very low correlation with clinical appearance. Luhmann *et al*^16^ noted that T1 tilt angle was not constant pre-operatively and post-operatively. Therefore, it was not used as a factor to evaluate the shoulder balance intra-operatively, except for Lenke III and VI (both had an unstructured proximal thoracic cure).

Based on the concept of the difference between the inner and outer shoulder heights of Qiu *et al*^8^, Ono *et al*^17^ confirmed that the phenomenon of “inner shoulder” imbalance is completely different from the conventional concept of “outer shoulder” imbalance. The authors suggested that the “inner shoulder” imbalance reflected the disequilibrium of the scapula (the trapezius muscle) and was the main correlation to spinal deformity due to tilt of the first rib and tilt of the T1 vertebra. When studying 89 patients with AIS, Kwan *et al*^11^ confirmed that neck tilt (with inner shoulder tilt) and shoulder tilt (outer shoulder tilt) were two separate phenomena and had very weak correlation to each other clinically. Neck tilt had a high moderate correlation with T1 tilt angle, while shoulder deviation had a high moderate correlation with CHD, CA, and CRID.

In this study, we recognised that the proportion of patients (26.0%) with severe SHo (>2cm) was much higher than the severe SHi proportion (6.0%) (Table I); thus, SHo was more clinically variable than SHi.

Our study recorded that neck tilt ≥5° accounted for 18% (9/50 patients). The rate of neck tilt was much lower than 40.2% (41/102 patients) of Yang *et al*^18^, who studied 102 patients with AIS who underwent surgery. This difference may be due to the AIS group consisting of only Lenke I and II in the study by Yang *et al*^18^. However, all suggest that a neck imbalance should be considered during surgery. Table II also shows that the neck tilt angle correlates with SHi rather than SHo.

**Fig 5:**
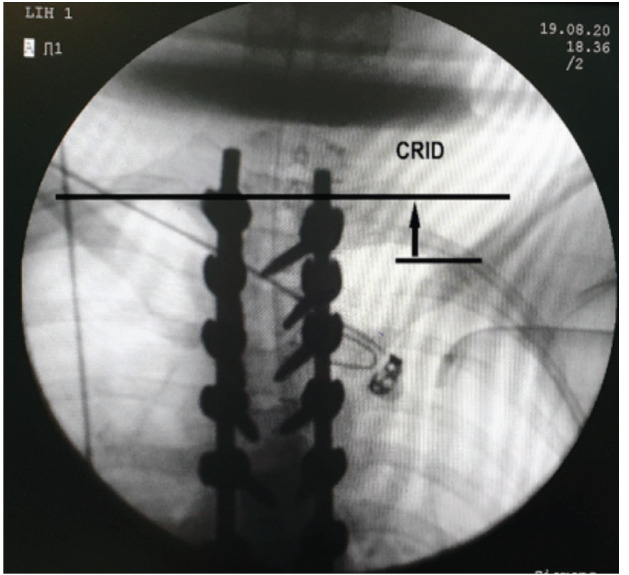
Measurement of Clavicle rib intersection distance (CRID) on image intensifier.

When evaluating the correlation index between clinical and radiographic shoulder balance in children with a double thoracic curve, Qiu *et al*^8^ noted that SHo had a high moderate correlation with CHD (r=0.67), CA (r=0.73), and CRID (r=0.74). This was like to our results according to (Table IV) (r=0.60, 0.63, and 0.72, respectively); so, CRID was most reliable among these 3 indices. Table IV also shows CCAD had a very-high correlation with SHi (r=0.84) and SHo (r=0.89); this result reinforces the retrospective study by Yagi *et al*^10^ that enrolled 85 patients with AIS. Their study proposed the CCAD index and demonstrated a very-high correlation between this index and shoulder girdle height difference post-operatively (odds ratio = 5.10; P=0.01). On the other hand, CCAD is difficult to assess quickly on radiography during operation. CA is easier to assess. This has been confirmed by Kuklo *et al*^19^; they suggested that CA was a pre-operative radiographic index to predict postoperative shoulder balance. However, CRID is more reliable, defined in cm rather than degree, able to be evaluated on image intensifier (Fig. 5); if shoulder balance is involved during surgery in operating room for patients with AIS, CRID index could be used to assess shoulder balance on the operating table. Moreover, the correlation coefficients between clinical and radiographic shoulder balance according to sex, BMI, type of main curve, and severity of main curve did not change significantly in this study.

Our study also found that the neck tilt angle had a high moderate correlation with T1 tilt angle (r=0.76); the correlation did not change significantly according to sex, BMI, type of main curve, and severity of main curve (Table IX). Meanwhile, Table IV showed a moderate correlation between SHo and the T1 tilt angle (r=0.43). Therefore, a radiographic image of the T1 tilt angle, which is the easiest to evaluate on radiography during operation, can be used to assess neck balance more reliably than assessing shoulder balance. This reinforces the conclusion of Kwan *et al*^11^ that neck imbalance has a very high correlation with the T1 tilt angle. Furthermore, the study of Chiu *et al*^20^ of 50 AIS patients followed at least 2 years post-operatively showed that the T1 tilt angle was the most reliable over time (r=0.78) among indices in the evaluation of shoulder balance and neck balance.

However, our study had a few cases and only focus on preoperative evaluation; so, it did not show if the correlation between clinical and radiographic shoulder and neck balance would be concerned with volume of curve reduction, upper implanted vertebra, difference between lying on operating table and standing images, as well as post-operative remodelling of this balance. Otherwise, no inter-observer assessment was also the limitation of this study.

## Conclusion

There was a very high correlation between SHo (shoulder tilt) and CCAD (r=0.89); the correlation between SHo and CRID was high-moderate (r=0.72), but CRID is easier than CCAD to evaluate on radiographs. On the other hand, T1 tilt angle, which is the easiest radiographic parameter to evaluate, had a high-moderate correlation with neck tilt angle (r=0.76) but a moderate correlation with SHo (r=0.43).
